# Widespread global peatland establishment and persistence over the last 130,000 y

**DOI:** 10.1073/pnas.1813305116

**Published:** 2019-02-25

**Authors:** Claire C. Treat, Thomas Kleinen, Nils Broothaerts, April S. Dalton, René Dommain, Thomas A. Douglas, Judith Z. Drexler, Sarah A. Finkelstein, Guido Grosse, Geoffrey Hope, Jack Hutchings, Miriam C. Jones, Peter Kuhry, Terri Lacourse, Outi Lähteenoja, Julie Loisel, Bastiaan Notebaert, Richard J. Payne, Dorothy M. Peteet, A. Britta K. Sannel, Jonathan M. Stelling, Jens Strauss, Graeme T. Swindles, Julie Talbot, Charles Tarnocai, Gert Verstraeten, Christopher J. Williams, Zhengyu Xia, Zicheng Yu, Minna Väliranta, Martina Hättestrand, Helena Alexanderson, Victor Brovkin

**Affiliations:** ^a^Department of Environmental and Biological Sciences, University of Eastern Finland, 70211 Kuopio, Finland;; ^b^Land in the Earth System, Max Planck Institute for Meteorology, 20146 Hamburg, Germany;; ^c^Department of Earth and Environmental Sciences, Division of Geography and Tourism, KU Leuven, B-3001 Leuven, Belgium;; ^d^Department of Earth Sciences, University of Toronto, Toronto, ON, M5S 3B1 Canada;; ^e^Institute of Geosciences, University of Potsdam, 14476 Potsdam, Germany;; ^f^Department of Anthropology, Smithsonian Institution, National Museum of Natural History, Washington, DC 20560;; ^g^Biogeochemical Sciences Branch, US Army Cold Regions Research and Engineering Laboratory, Fort Wainwright, AK 99703;; ^h^California Water Science Center, U.S. Geological Survey, Sacramento, CA 95819;; ^i^Permafrost Research Section, Geosciences Department, Alfred Wegener Institute Helmholtz Centre for Polar and Marine Research, 14473 Potsdam, Germany;; ^j^College Asia and the Pacific, Australian National University, Canberra, ACT 2600, Australia;; ^k^Department of Geological Sciences, University of Florida, Gainesville, FL 32611;; ^l^Eastern Geology and Paleoclimate Science Center, U.S. Geological Survey, Reston, VA 20192;; ^m^Department of Physical Geography, Stockholm University, 10691 Stockholm, Sweden;; ^n^Department of Biology, University of Victoria, Victoria, BC, V8W 2Y2 Canada;; ^o^School of Life Sciences, Arizona State University, Tempe, AZ 85287;; ^p^Department of Geography, Texas A&M University, College Station, TX 77843;; ^q^Environment and Geography, University of York, YO105DD York, United Kingdom;; ^r^Department of Zoology and Ecology, Penza State University, 440026 Penza, Russia;; ^s^Goddard Institute for Space Studies, NASA, New York, NY 10025;; ^t^Department of Earth and Environmental Sciences, Lehigh University, Bethlehem, PA 18015;; ^u^School of Geography, University of Leeds, LS2 9JT Leeds, United Kingdom;; ^v^Department of Geography, University of Montreal, Montreal, QC, H2V 2B8 Canada;; ^w^Research Branch, Agriculture and Agri-Food Canada, Ottawa, ON, K1A 0C6 Canada;; ^x^Department of Earth and Environment, Franklin and Marshall College, Lancaster, PA 17603;; ^y^Institute for Peat and Mire Research, School of Geographical Sciences, Northeast Normal University, 130024 Changchun, China;; ^z^Environmental Change Research Unit, Ecosystems and Environment Research Programme, University of Helsinki, 00014 Helsinki, Finland;; ^aa^Department of Geology, Lund University, 223 62 Lund, Sweden

**Keywords:** peatlands, carbon, methane, carbon burial, Quaternary

## Abstract

Glacial−interglacial variations in CO_2_ and methane in polar ice cores have been attributed, in part, to changes in global wetland extent, but the wetland distribution before the Last Glacial Maximum (LGM, 21 ka to 18 ka) remains virtually unknown. We present a study of global peatland extent and carbon (C) stocks through the last glacial cycle (130 ka to present) using a newly compiled database of 1,063 detailed stratigraphic records of peat deposits buried by mineral sediments, as well as a global peatland model. Quantitative agreement between modeling and observations shows extensive peat accumulation before the LGM in northern latitudes (>40°N), particularly during warmer periods including the last interglacial (130 ka to 116 ka, MIS 5e) and the interstadial (57 ka to 29 ka, MIS 3). During cooling periods of glacial advance and permafrost formation, the burial of northern peatlands by glaciers and mineral sediments decreased active peatland extent, thickness, and modeled C stocks by 70 to 90% from warmer times. Tropical peatland extent and C stocks show little temporal variation throughout the study period. While the increased burial of northern peats was correlated with cooling periods, the burial of tropical peat was predominately driven by changes in sea level and regional hydrology. Peat burial by mineral sediments represents a mechanism for long-term terrestrial C storage in the Earth system. These results show that northern peatlands accumulate significant C stocks during warmer times, indicating their potential for C sequestration during the warming Anthropocene.

## Significance

During the Holocene (11,600 y ago to present), northern peatlands accumulated significant C stocks over millennia. However, virtually nothing is known about peatlands that are no longer in the landscape, including ones formed prior to the Holocene: Where were they, when did they form, and why did they disappear? We used records of peatlands buried by mineral sediments for a reconstruction of peat-forming wetlands for the past 130,000 y. Northern peatlands expanded across high latitudes during warm periods and were buried during periods of glacial advance in northern latitudes. Thus, peat accumulation and burial represent a key long-term C storage mechanism in the Earth system.

The distribution of carbon stocks during glacial cycles represents a key uncertainty in the long-term global C budget and the global climate system (1, 2). During the last glaciation, ice core records show low atmospheric CO_2_ concentrations and a strong increase following deglaciation, correlating with temperature increases. However, the mechanisms behind these observations are still unknown; hypotheses include both marine (1) and terrestrial processes (2, 3). At present, northern peatlands, wetlands with thick (>30 cm to 40 cm) organic sediments, contain an estimated 400 Pg C to 500 Pg C (4, 5), and tropical peatlands contain an estimated ∼105 Pg C (4, 6). These peatlands have sequestered atmospheric CO_2_ over millennia because plant productivity exceeds decomposition, which is slowed by the saturated and anoxic soil conditions found in these wetlands and leads to the accumulation of undecomposed organic matter (peat). As the largest natural source of methane (CH_4_) to the atmosphere (7), tropical and high-latitude wetland emissions are often invoked to explain variations in atmospheric CH_4_ concentrations over glacial cycles (8) and abrupt CH_4_ increases during periods of rapid climatic change (8, 9).

**Fig. 1.**
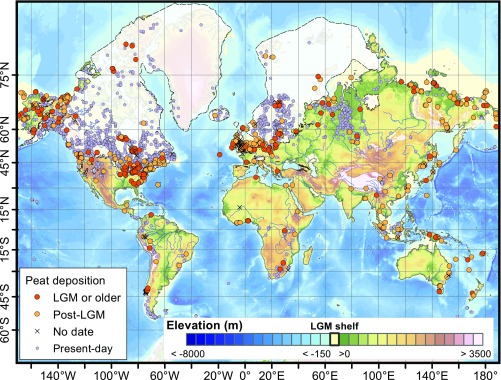
Locations of buried peat and present-day peatland sites; buried peat profiles from the LGM (18 ka) and before (orange circles), post-LGM (yellow circles), and profiles without chronological control (black crosses), and basal ages from present-day peatlands (purple circles). North American/Greenland and Scandinavian ice sheet extents are shown by white area with dashed border (44), exposed continental shelf areas during the LGM (yellow) are based on Etopo DEM + Bathymetry using a −125-m sea level (45). Overlapping crosses and circles indicate multiple profiles with and without chronological control.

Beyond CH_4_ emissions, the role of peatlands in the global C cycle on glacial−interglacial time scales has not been considered, due to a lack of systematic evidence of peatland extent before the Last Glacial Maximum (LGM, 21 ka to 18 ka; Fig. 1). Previous studies have explored the timing and locations of peatland formation (or “peat initiation”) and expansion in northern high latitudes during the Holocene (from 11.6 ka to the preindustrial period) using basal ages, the oldest age of the deepest sediments found in present-day peatlands. These studies have shown that most peatlands formed following the LGM (9–13). On the other hand, large coal deposits from the Carboniferous period (359 Ma to 299 Ma) and the Miocene (23 Ma to 5 Ma) indicate that significant peat accumulated before the Holocene, but little is known about peat deposits during the Quaternary (2.58 Ma to 12 ka) despite modeling studies showing the likely importance of a peatland C pool in the global C cycle (14).

Here, we identify the spatial and temporal distribution of ancient peatlands preserved by burial under minerogenic sediments (“buried peat deposits”) and model global peatland C stocks for the past 130,000 y (130 ka) to test the response of peatland C stocks to the highly variable climate conditions before the Holocene. We create a synthesis dataset of buried peat deposits that includes 1,063 profiles globally (Fig. 1), including 37 previously unpublished profiles, by compiling data from sediment exposures and soil, lake, and marine cores containing peat sections (*Materials and Methods* and Dataset S1). In addition to location, we use several attributes of buried peat deposits in our analysis: (*i*) the timing of active peat accumulation when sites were actively accumulating peat (determined from sediment dating methods) as opposed to being buried or otherwise inactive; (*ii*) the number of sites that were actively accumulating peat at the same time (active buried sites, a count), which is a proxy for peatland extent; and (*iii*) the thickness of these buried peat deposits, which is a proxy for the total C stock of the peat deposits that accumulated during the period of active deposition (thickness, when reported). We model peatland C stocks from 126 ka to the preindustrial (1850 CE) using an Earth System Model of Intermediate Complexity coupled to a higher resolution Dynamic Global Vegetation Model (15) (*Materials and Methods*).

## Results and Discussion

### Spatial and Temporal Distribution of Peats in the Northern Region (>40°N).

**Fig. 2.**
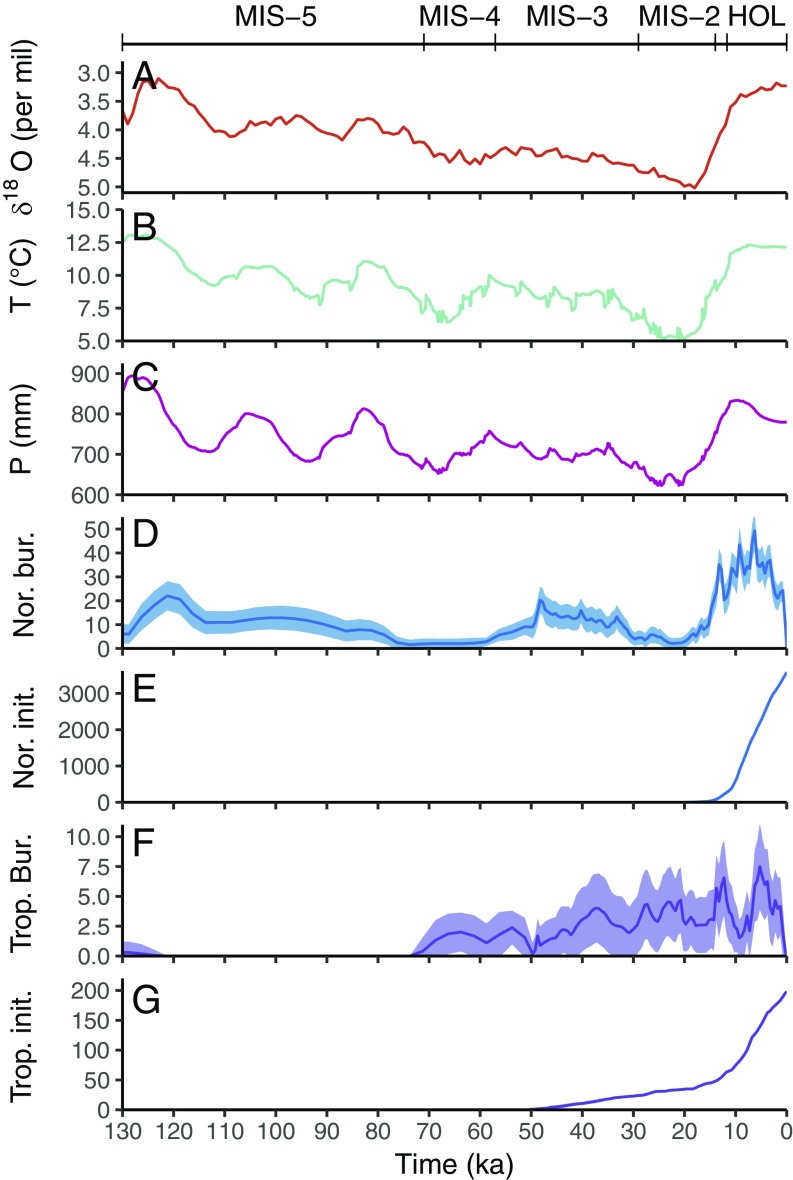
Climate boundary conditions and peat formation records for northern (Nor.; >40°N) and tropical (Trop.; 30°N to 30°S) peatlands for the last 130 ka. At the top are corresponding names for chronostratigraphic units used in the text, including the Holocene (HOL). (*A*) LR04 δ^18^O stack (38); (*B*) simulated mean annual temperature for global land areas (39); (*C*) simulated annual precipitation for global land areas (39); (*D*) number of active northern peat deposits now buried (count); (*E*) northern peatland initiation (count); (*F*) number of active peat deposits (now buried) in tropical regions (count); and (*G*) tropical peatland initiation (count).

There is substantial evidence for widespread northern peatlands from more than 40 sites during the last interglacial (130 ka to 116 ka, MIS 5e), when continental ice sheets were largely absent in the Northern Hemisphere (Fig. 2*D* and *SI Appendix*, Fig. S1). During a period of cooling during MIS 4 (∼71 ka BP to 57 ka BP; Fig. 2*B*), northern buried peat records decreased by 75% to the smallest number outside of the LGM (Fig. 2*D*, Table 1, and *SI Appendix*, Fig. S1). As temperatures increased during MIS 3 (57 ka to 29 ka), the number of northern buried peat records increased sixfold, particularly between 57 ka and 45 ka (Fig. 2*D* and Table 1). Peatland expansion continued between 35 ka and 29 ka with peat formation in the northern coastal lowlands of Siberia, Alaska and Beringia, and central North America (*SI Appendix*, Fig. S1).

**Table 1.** Summary of northern (>40° N) peatland sites and modeled active C stocks between the last interglacial (130 ka) and the LGM (18 ka)

Period	Age, ka	Active buried sites, count	Thickness, cm	Modeled active C stock, Pg
MIS 5e	130–116	45	70 (50–150)	280 (215–405)
MIS 5a−d	116–71	49	75 (50–140)	260 (200–380)
MIS 4	71–57	17	90 (40–340)	210 (160–305)
MIS 3	57–29	120	100 (40–200)	265 (205–385)
MIS 2	29–21	23	65 (40–100)	135 (105–195)
LGM	21–18	11	25 (20–110)	80 (60–115)

“Active buried sites” indicates the total number of observed sites with active peat accumulation, the median observed peat thickness in the present day (25th and 75th percentile ranges shown in parentheses), and the modeled active peatland C stock (model error shown in parentheses). The correlation between active sites and modeled C stocks was *ρ* = 0.77 using Spearman’s rank correlation.

After 29 ka, the number of active northern peat deposits decreased by >80% (Fig. 2*D* and Table 1), coinciding with a cooling trend in Northern Hemisphere temperature (Fig. 2 *A* and *B*), the expansion of glaciers and ice sheets (Fig. 2*A*), and the burial of peat by glacial sediments. Even in nonglaciated regions of Siberia, Alaska, and southeastern United States, active peatland extent was greatly reduced (Table 1 and *SI Appendix*, Fig. S1) as peats not covered by glacial sediments were buried by aeolian deposits (27%), coastal sediments (30%), and permafrost-associated deposits (16%). The number of active northern peat deposits reached a minimum during the LGM (Table 1 and Fig. 2*D*) as temperatures reached their minimum (Fig. 2*B*) and ice extent reached its maximum (Fig. 2*A*). Eighty percent of the remaining peat records at the LGM were found in present-day coastal zones, while limited peatland formation also occurred at the southern margin of glaciated regions (*SI Appendix*, Fig. S1).

As glacial retreat began after 18 ka, peatlands expanded northward in newly exposed lowland areas along the southern ice margins of the Laurentide and Scandinavian ice sheets, forming both now-buried peats and present-day peatlands (Fig. 2*D*, Table 2, and *SI Appendix*, Fig. S2). The rapid establishment of northern peatlands occurred during the first half of the Holocene (Table 2) as peat accumulated in the West Siberian Lowlands, Fennoscandia, and western Canada. The deposition of now-buried peats also increased significantly following the onset of the Holocene, but decreased after 5 ka (Fig. 2*D* and Table 2) as coastal areas flooded (52% of sites) or hydrological conditions changed (30% of sites).

**Table 2.** Summary of northern and tropical peatland records since the LGM

		Northern				Tropical		
	Age, ka	Active buried, count	Present day		Modeled C stocks, Pg	Active buried, count	Present day	
Period			Count	Percent			Count	Percent
LGM	21–18	11	6	0.2	80 (60–120)	11	37	20
Bølling−Allerød	14.7–12.7	84	209	5.8	110 (85–160)	17	57	30
Holocene	11.7	41	328	9.1	140 (110–205)	10	65	33
	8.2	50	1,375	38.3	225 (170–325)	5	96	50
Mid-Holocene	5	48	2,387	66.6	305 (235–440)	13	146	75
Present day	2000 CE	0	3,586	100	410 (315–590)*	0	197	100

For both northern and tropical peat sites, the number of now-buried sites with active peat deposition is given (“Active buried”), as well as the cumulative number and percentage of present-day peatland sites that were established by the period of interest (“Present day”). Modeled active northern peatland C stocks are also shown and correlated well with total northern peat sites (active buried + present day; *r* = 0.99); active tropical peatland C stocks are shown in *SI Appendix*, Table S1. Modeled C stocks model error shown in parentheses.

* Modeled C stocks are from preindustrial period (0.1 ka). Since the preindustrial period, peatland harvesting, drainage, and other land use factors have been observed (25) but are not modeled.

Modeled northern peatland C stocks agreed well with observations of active peat accumulation in now-buried peat deposits before the LGM (Table 1; *ρ* = 0.77). During MIS 5e, the maximum modeled active northern peatland C stocks were 340 Pg at 120 ka, corresponding to the largest number of northern sites with active peat deposition before the LGM (Fig. 2*D*). During MIS 4, modeled active peatland C stocks decreased to 210 Pg C, corresponding to a decrease in peatland extent, here evidenced by the number of sites with active peat deposition (Table 1). During warmer MIS 3, modeled active peatland stocks again increased to 265 Pg C, corresponding to a significant increase in peatland extent (Table 1). As glaciers expanded during MIS 2 and into the LGM, modeled active peatland C stocks decreased by 70% from MIS 3 values to a minimum of ∼80 Pg C. During this period, active peatland extent decreased significantly as peats were buried by glacial sediments and other sediment types; observations show that the remaining peats were shallower (Table 1).

Following the LGM, modeled active peatland C stocks increased slowly before the Holocene, adding ∼60 Pg C, which correlates well with the slow increase in active peatland formation observed during this period (Table 2). During the beginning of the Holocene, modeled active peatland C stocks increased rapidly, corresponding to the strong increase in observed peatland initiation (Fig. 2*E* and Table 2; *r* = 0.99). A significant number (33%) of present-day peatlands were formed after 5 ka (Fig. 2*E* and Table 2), and modeled active peatland C stocks increased by approximately the same amount (34%) during this period. Modeled active northern peatland C stocks reached a maximum of 410 Pg C in the preindustrial period (315 Pg C to 590 Pg C; Table 2), an increase of 330 Pg C since the LGM.

### Spatial and Temporal Distribution of Peats in the Tropics (30°N to 30°S).

The first known buried peat deposit from the tropics formed between 164 ka and 122 ka (16) in New Guinea, followed by a hiatus with no evidence of tropical peat deposition until 60 ka (Fig. 2*F* and *SI Appendix*, Fig. S3). The first evidence of peatland establishment in equatorial and southern Africa dates to 50 ka to 45 ka (*SI Appendix*, Fig. S3); the majority of sites from that time persist to the present day (*SI Appendix*, Fig. S3 and Dataset S2). The number of actively accumulating peats in the tropics increased after 45 ka, then decreased during MIS 2 through the LGM as peats were buried by fluvial and coastal processes (*SI Appendix*, Table S1 and Dataset S1). However, the formation of new peatlands resulted in little apparent change in tropical peatland distribution (*SI Appendix*, Fig. S3).

As global temperatures increased after the LGM and into the Bølling−Allerød, the rate of peatland initiation increased after ∼15 ka for both buried and present-day tropical peatlands (Fig. 2*G* and Table 2) as peats accumulated on the then-exposed continental shelves in Indonesia and western Africa (*SI Appendix*, Fig. S4). The number of active tropical peat records decreased and peat initiation slowed between the Bølling−Allerød and early Holocene (Fig. 2*F* and Table 2) as continental shelves flooded and buried coastal sites in Southeast Asia, including sites in the Strait of Malacca, Thailand coast, and Java Sea (*SI Appendix*, Fig. S4). As the sea level stabilized (17), the number of now-buried tropical peat records in Southeast Asia more than doubled during the mid-Holocene between 8.2 ka and 5 ka (Fig. 2*F*, Table 2, and *SI Appendix*, Fig. S4) as peatlands expanded across Indonesia and Malaysia (Fig. 2*G*, Table 2, and *SI Appendix*, Fig. S4). While the areas of active tropical peatland formation shifted in space and time (*SI Appendix*, Figs. S3 and S4), the total modeled active tropical peatland C stocks remained relatively constant throughout the interglacial at an estimated 145 Pg C (80 Pg C to 215 Pg C; *SI Appendix*, Table S1).

### Factors Controlling the Distribution of Peat in Space and Time.

Peat accumulation occurs when vegetation productivity exceeds decomposition losses and is facilitated by anoxic conditions due to poor drainage in wetlands. Understanding the drivers of peat accumulation and loss under a broad range of climatic conditions can ultimately improve projections of the response of peatland C stocks to future climatic changes (18) through improved representation of processes controlling peat accumulation. Process-based modeling approaches have predicted a wide range of outcomes in response to future climate change, from substantial loss of peat due to drying (19) and permafrost thaw (20) to continued peat accumulation (21). These data show another possible fate for peat: burial of peat by mineral sediments.

Our results show that warm periods with higher precipitation (e.g., MIS 5e, MIS 3, and Holocene) corresponded to a higher occurrence of northern peat deposition and greater northern peatland C stocks, evidenced by the observed number of sites, observed peat thickness, and modeled C stocks (Fig. 2 *B*, *D*, and *E* and Table 1). Whether increased peat formation during warm periods was caused by changes in productivity and decomposition rates or other factors such as increases in area is unclear. A recent analysis suggests that the number of growing degree days is the key driver of northern peatland formation in ice-free areas during the Holocene (13). However, higher temperatures also correlate with smaller areal extent of ice sheets and glaciated areas (Fig. 2 *A* and *B*), potentially exposing relatively flat, vegetation-free terrain and alleviating a spatial bottleneck for peatland formation (10). Peat formation on formerly glaciated and ice sheet areas was responsible for ∼30% of the modeled increase in peatland areas between the LGM and the preindustrial Holocene (*SI Appendix*, Table S2). Regardless, net peat accumulation will likely continue in topographically favorable areas with warming as long as disturbances such as wildfire, drainage, or flooding are not significant (18).

Northern peatland extent and C stocks were smallest during cold, dry periods with enhanced glaciation (e.g., MIS 4 and MIS 2, or 71 ka to 57 ka and 29 ka to 21 ka, respectively; Fig. 2, Table 1, and *SI Appendix*, Fig. S1). Colder periods may not have directly resulted in the loss of peat (e.g., to the atmosphere), but instead favored processes (aeolian, glacial, and glaciofluvial) that resulted in rapid mineral deposition and subsequent peat burial while limiting new peatland development or recovery of peat accumulation due to dry or continental conditions (22). While limited observational evidence of peats during these cold periods does not mean peatlands were absent, the persistence of peat deposits from older, warmer periods (MIS 3 and MIS 5e; Fig. 2*D*) indicates this trend of increased peat formation during warmer times and burial during colder periods is robust.

During warm periods (the Holocene) and in warm locations (the tropics), peat burial was related to factors other than temperature. Tropical peatland deposition was relatively insensitive to global temperature fluctuations, as evidenced by the persistent presence of tropical peatlands on the landscape after 50 ka during a range of climatic conditions in both data and model results (Fig. 2*F*, Table 2, and *SI Appendix*, Table S1). Instead, tropical peat formation responded mainly to changing hydrologic conditions. For example, approximately one-third of the tropical buried peats that formed during the Holocene were buried as sea level rose (15/46 sites), while others were formed as rising sea level altered regional hydrology in coastal regions (13, 17). Hydrological changes were responsible for the cessation of peat accumulation at approximately one-third of now-buried tropical peatland sites during the Holocene (14/46 sites) as water tables in lakes and wetlands both rose and fell. Similar patterns were observed for northern peats after 5 ka, when coastal flooding and changing hydrology buried >80% of the buried peat sites. Additionally, anthropogenic influence was important for the burial of tropical peatlands (9/46 sites) and some northern peatlands (2/411 sites) during the Holocene (23). The burial and destruction of peats in Central America, New Guinea, and Borneo have been attributed to a combination of changes in agricultural practices, deforestation, and changing environmental conditions (24). In western and central Europe, anthropogenic factors such as changing agricultural practices, deforestation, and subsequent changes in hydrology and soil erosion led to an increase in floodplain sedimentation and peat burial (25) in many peat-forming wetlands located in floodplains.

### Implications for the Global C Budget.

While the importance of northern peatland expansion for global C cycles during the Holocene has been previously recognized (2, 4, 14, 26), these new results show the importance of both tropical and northern peatlands to the global C cycle during and before the Holocene. The accumulation of 560 Pg C in peatlands globally comprises between 18% and 25% of the total land C modeled by LPJ for the preindustrial Holocene and represents a significant C storage term in the Earth system. From the LGM to preindustrial, the global peatland C stock increased by 300 Pg C to 330 Pg C, in agreement with previous estimates of increases in histosol and peat C storage from the LGM to the present (3, 27). The increase in peatland C was substantially larger than the 190-Pg C increase in the atmospheric CO_2_ inventory between the LGM and the preindustrial period. To balance the global C budget for the LGM to the preindustrial period, the remainder of the C budget change must have been supplied by other C pools, likely the ocean.

Previously, it has been assumed that the loss of peatlands meant increased decomposition and release of peatland C to the atmosphere (19, 28), but these data demonstrate otherwise. With burial by mineral sediments, peat C can be incorporated into long-term C storage in sediment, as evidenced by the age of these deposits (Figs. 1 and 2 and *SI Appendix*, Fig. S1). While decomposition of buried peat may occur, this may be limited in deep soils and subglacial sediments by anoxia resulting from slow rates of oxygen diffusion or saturation (29), limited microbial activity at depth (30), and cold temperatures or permafrost (31). Peat accumulation and subsequent burial by mineral sediments provides a mechanism for the transfer of atmospheric CO_2_ to a stable terrestrial C pool, where it can be preserved for millennia or longer despite decreases in active peatland area and C stocks (Fig. 2 and Table 1).

Peatland C accumulation and burial has potential implications for the redistribution of C among global reservoirs at glacial/interglacial time scales, which has been a long-standing debate (1, 2). Proposed mechanisms for CO_2_ sequestration during the LGM include enhanced CO_2_ storage in deep oceans (1) or formation of inactive terrestrial C stocks (2), such as C buried by glacial sediments (32) or permafrost soils (2, 3, 26, 33). These observational data demonstrate that peat burial by mineral sediments was widespread during the glacial expansion preceding the LGM (Fig. 2*D* and Table 1) and provide an alternative explanation for the incorporation of significant amounts of organic matter into long-term terrestrial sediment C stocks and permafrost before the LGM (2, 3). Our modeling results can be used to estimate the upper bounds of peat C burial from the loss of active peatland C stocks between MIS 3 and the LGM, assuming all peat was buried rather than lost to the atmosphere. These model results show maximum total global peatland C stocks of 433 Pg C and 260 Pg C for MIS 3 and the LGM, respectively, a decrease of 170 Pg C in active peatland C stocks. The loss of active peat C is substantially larger than the ∼30 Pg C decrease in the atmospheric CO_2_ inventory during this period. To balance the global C budget for MIS 3 to the LGM, the remainder of the C budget change must to have been taken up by other C pools, likely the ocean. In the scenario where all buried peat C was preserved in the subsoil rather than lost to the atmosphere, a buried peat C stock of 170 Pg C requires much less C uptake by the ocean and other pools. Previously, Ciais et al. (2) hypothesized an increase of ∼700 Pg C in inert land C at the LGM compared with the preindustrial Holocene. These results show that this change cannot be linked to active peatlands, which were at a minimum during the LGM (Fig. 2 and Table 1). Peat burial and subsequent loss could explain part of the inert land C change but would require substantial contributions from other terrestrial environments and processes (3). More information on peat properties as well as process-based scenario modeling will be required to better constrain the size of both buried peat C pools and terrestrial C pools during this climatic transitional period.

These observations of buried peats demonstrate that peatlands have been an important C stock since the last interglacial (Table 1). In particular, actively forming northern peatlands both accumulated C and emitted CH_4_ during warm periods. During colder periods of glacial advance, the burial of significant northern peat C stocks by mineral sediments and formation of permafrost would have all but stopped decomposition and CH_4_ emissions (34), resulting in the long-term burial of peatland C. The widespread distribution of buried peats and the large magnitude of the change in peatland C stock throughout the last glacial cycle suggests that peat formation during warmer times and burial during colder periods has a significant impact on the global carbon cycle that has not been previously quantified (2).

## Materials and Methods

### Buried Peat Dataset.

We compiled 1,063 records of buried peat layers from peats overlain by minerogenic sediments described in the published literature and from 37 unpublished profiles (Fig. 1 and Dataset S1). We identified profiles based on author knowledge, solicitations through existing research networks, and literature searches on Web of Knowledge and Google Scholar using the terms “buried peat,” “buried peat deposits,” “histic paleosols,” “organic paleosols,” “interglacial peat,” “MIS 5 and peat,” and “MIS 3 and peat.” We defined peat broadly as organic-rich sediment derived from wetland or limnic environments deposited in situ or within the local catchment. We extracted information on the profile location, depth of the organic-rich sediments, the timing of deposition (when available), the depositional environment of the organic-rich sediment (alluvial, limnic, wetland, or upland), the type and origin of the overlying sediments, and other site descriptors. The dataset is publicly available via the PANGAEA data archive (35).

Chronological control for the timing of peat formation was available for 930 profiles (88% of samples) and was based mainly on calibrated radiocarbon dates for 786 profiles younger than 50 ka (alternative dating was used for 18 profiles). For profiles older than 50 ka, chronologies were based on tephrochronology (14 profiles), optically or thermally stimulated luminescence dating (six profiles), stratigraphic position relative to tills and other sediment types of known depositional age (25 profiles, plus 19 profiles with infinite radiocarbon dates), pollen (11 profiles), or Uranium−Thorium dating (eight profiles). Multiple dating proxies were used at 41 profiles. The use of radiocarbon dating imposes some important considerations. Notably, the apparent increase in the number of buried peat sites after 50 ka (Fig. 2 *D* and *F*) is likely related to the technical limitations of radiocarbon dating, because deposits from <50 ka are more readily “datable” than older deposits. Other potential errors in the chronological control of this study include contamination by modern radiocarbon, ancient radiocarbon, and/or poor chronological constraints due to having only one date from within the buried peat section or proximate sediment layers (683 of 930 dated deposits) or lack of suitable materials for various dating approaches. Ideally, additional dating of buried peat sections would constrain the duration of peat persistence on the landscape and clarify the timing of peat development in relation to atmospheric CO_2_ and CH_4_ records.

We used peatland basal ages (oldest date from present-day peatlands, indicating the beginning of peat accumulation) to place the development of the buried peats in the context of the development of present-day peatlands. The peatland initiation dataset consisted of 3,942 basal ages and was based on a compilation of several existing basal age datasets for northern peatlands (9, 11, 12, 36) and tropical peatlands (4, 17), and 473 additional basal ages from newer literature not included in previous compilations (Dataset S2). The peatland initiation dataset is archived and publically available (35).

All radiocarbon ages were calibrated with IntCal13 (36), and all ages referred to in the text have been calibrated (cal BP). Calibrated dates were rounded to the nearest decade. Further details on the evaluation of chronologic uncertainty can be found in *SI Appendix*. Profiles without chronological control (133 of 1,063 profiles) remain in the database (Fig. 1) but could not be used to track peat C persistence over the last glacial cycle (Fig. 2 *D* and *F*). For comparison between peat records and climate (Fig. 2), we used the harmonized δ^18^O records (38), and the results of the CLIMBER2 Earth system model simulations through the last glacial cycle (39).

### Global Peatland Modeling.

We performed a transient model experiment using a climate−carbon cycle model, an updated version of the peatland-enabled CLIMBER2-LPJ model (15), to determine peatland extent and C stocks through the last glacial cycle, because these could not be interpreted from the observations (*SI Appendix*). Briefly, CLIMBER2-LPJ consists of the Dynamic Global Vegetation Model LPJ (40), coupled to the Earth System Model of Intermediate Complexity CLIMBER2 (41). LPJ is run on a 0.5° × 0.5° grid and is coupled to the coarser grid of CLIMBER2 via climatic anomalies and carbon fluxes (15, 42). Ice sheet areas, as well as sea level and isostasy, are prescribed from an experiment with an ice sheet-enabled version of the CLIMBER2 model (43). The global peatland model determines peatland location and extent from a combination of topography and grid cell-scale water balance using a TOPMODEL approach as described in Kleinen et al. (15), as opposed to being prescribed, as in other global model simulations of Holocene peatlands (21). This allows peatland areas to form dynamically in response to changing hydrologic conditions. Sea level is dynamic in this model framework, allowing us to estimate peatland areas on exposed continental shelves. The peatland model was driven with orbital changes, CO_2_ concentrations derived from ice core data, and ice sheet extent determined using an ice sheet-enabled version of the CLIMBER2 model (43). The model was initialized with a 5,000-y spin-up period under early Eemian boundary conditions at 126 ka BP and subsequently run transiently from 126 ka BP until 0 BP. Further details about model parameterization and evaluation can be found in *SI Appendix*.

## Supplementary Material

Supplementary File

Supplementary File

Supplementary File
