# Supplementary material for: Widespread global peatland establishment and persistence over the last 130,000 y
Source: Proc Natl Acad Sci U S A. 2019 Feb 25;116(11):4822–7. doi: 10.1073/pnas.1813305116 (PMC6421451; doi:10.1073/pnas.1813305116)
Supplement: Supplementary File [file pnas.1813305116.sapp.pdf]

PNAS
www.pnas.org

Supplementary Information for

Widespread global peatland establishment and persistence over the last 130,000 years

Claire C. Treat, Thomas Kleinen, Nils Broothaerts, April S. Dalton, René Dommain, Thomas A. Douglas, Judith Drexler, Sarah A. Finkelstein, Guido Grosse, Geoff Hope, Jack Hutchings, Miriam C. Jones, Peter Kuhry, Terri Lacourse, Outi Lähteenoja, Julie Loisel, Bastiaan Notebaert, Richard Payne, Dorothy Peteet, A. Britta K. Sannel, Jonathan M. Stelling, Jens Strauss, Graeme T. Swindles, Julie Talbot, Charles Tarnocai, Gert Verstraeten, Christopher J. Williams, Zhengyu Xia, Zicheng Yu, Minna Väliranta, Martina Hättestrand, Helena Alexanderson, Victor Brovkin

Claire Treat
Email: claire.treat@uef.fi

This PDF file includes:

Supplementary text
Figs. S1 to S7
Tables S1 to S3
References for SI reference citations

Other supplementary materials for this manuscript include the following:

Datasets S1 to S2

www.pnas.org/cgi/doi/10.1073/pnas.1813305116

Supplementary Information Text

Estimates of chronological uncertainties in observations.

In order to assess the uncertainties associated with the profile and peatland basal age chronologies, we used bootstrap resampling (n=1000) to develop an uncertainty distribution based on the chronologic uncertainty. For each statistical replicate, all buried peats samples were assigned ages based on randomly sampled values that fell within the maximum and minimum calibrated ages (including 2-sigma age uncertainty) for each deposit (r command: sample). If errors on the radiocarbon ages were not reported, we assumed a 10% error. A similar procedure was used for assessing error in peatland initiation dates. Calibrated peatland basal ages were assigned an age based on a randomly sampled value that fell within the 2-sigma range of the calibrated basal peat date; peat was assumed to occur thereafter. The resampling procedures were repeated 1000 times for each analysis (northern buried peats, northern peat initiation, tropical buried peats, tropical peatland initiation); the mean and 95% confidence intervals of the statistical replicates are shown.

Estimates of past and present global peatland carbon stocks.

Approaches for estimating present-day global C stocks generally follow one of two approaches. First, a potential peatland domain is mapped. Carbon stocks are estimated by multiplying the peatland areas by estimates of peat depth multiplied by a mean C density (e.g. 1). The alternative approach estimates C stocks using the area and time of peatland initiation (or basal age) to calculate a time series of peatland area, which is then multiplied by a time series of apparent C accumulation rates (2). This approach has the advantage of providing some constraint on peatland C stocks during the Holocene, although actual stocks likely differed significantly (3).

These approaches are limited in the potential to reconstruct peatland C stocks prior to and during the Holocene for several reasons. Existing data-based reconstructions require and assume a net deposition of peat, while our buried peat database demonstrates that peat accumulation can end. In these approaches, there is no mechanistic, process-based representation of peat accumulation and decomposition, which limits the analysis of the response of peatland extent, thickness, and C stocks to changing climatic conditions, which can include a net loss of C (4). Additionally, data-based reconstructions rely on maps of present-day peatland area and do not account for the dynamics of peatland areas, while our dataset shows that peatlands were present in larger or different areas in the past than present day (Figs. S1-4).

Our dataset provides point-based evidence of peatland presence in the past, with some measurements of present-day peat thickness and organic matter content. However, without information on spatial extent or carbon density of these buried peats, it is impossible to estimate the C stocks. From macrofossil analysis in numerous samples, we know that many of these profiles are remnants of past peatlands. Estimating possible or probable C stocks in present and past buried peats requires significant assumptions about peatland extent, present-day and past C densities, and decomposition trajectories (e.g. initial peat mass vs. peat mass remaining). However, there is very limited geochemical data from the buried peat profiles to support assumptions of C density at this time (Data S1), especially given effects such as compaction due to overlying sediments. Few records (< 5%) contained information about organic matter or organic carbon content, so we are not able to definitively evaluate whether these organic-rich sediments could be classified as peat using the strict definition for histosols (peat) employed by soil scientists (5). Many lithological and stratigraphic descriptions of soil profiles simply use the term "peat" to describe sediments with a high organic content; acknowledgement of the differences in terminology and methodology between soil scientists and stratigraphers dates back more than 25 years (6). In addition, compaction of peat deposits by the subsequent deposition of overlying mineral sediments permanently increases peat bulk density and decreases peat thickness, making it nearly impossible to determine original peat thickness.

Uncertainties around peatland areas in the past are even more extensive than uncertainties in C density, especially prior to the Holocene. To account for these factors, we used a process-based peatland model to estimate C stocks since the last interglacial. The model uses dynamic wetland areas based on local hydrology, topography, and ice sheet extent and calculates net carbon accumulation as the balance between productivity and decomposition.

Model experiment: Peatland C stocks.

We performed a transient model experiment using a climate-carbon cycle model, an updated version of the peatland-enabled CLIMBER2-LPJ model (7) in order to determine peatland extent and C stocks through the last glacial cycle. Briefly, CLIMBER2-LPJ consists of the dynamic Global Vegetation Model LPJ (8), coupled to the Earth System Model of Intermediate Complexity CLIMBER2 (9). LPJ is run on a 0.5°x0.5° grid and is coupled to the coarser grid of CLIMBER2 via climatic anomalies and carbon fluxes (7, 10). Ice sheet areas, as well as sea level and isostasy, are prescribed from an experiment with an ice-sheet enabled version of the CLIMBER2 model (11). The land-sea mask of the model is dynamic, with the land area at any time determined from topography, taking into account bedrock depression and sea level. Newly deglaciated points, as well as newly formed land points on the continental shelves, are initialized as bare ground, with the LPJ vegetation dynamics determining the subsequent development of vegetation and peatland cover, while newly glaciated points and grid points flooded due to sea level rise are removed from the land domain.

The global peatland model determines peatland location and extent from a combination of topography and grid-cell scale water balance using a TOPMODEL approach as described in Kleinen, Brovkin and Schuldt (7), as opposed to being prescribed, as in other global model simulations of Holocene peatlands (12). This allows

peatland areas to form dynamically in response to changing hydrologic conditions. Sea level is dynamic in this model framework, allowing us to estimate peatland areas on exposed continental shelves.

The peatland model was originally developed and calibrated for northern peatlands. Net carbon accumulation is calculated as the difference between productivity and decomposition, which occurs aerobically above the water table and anaerobically below. Here, for the first time, we model both the tropical and the northern extra-tropical regions as well as peatland development on continental shelves. This required several modifications to the published peatland model setup (7), including small changes to calculations of peatland extent and decomposition rates. First, the peatland-extent module was set up for the global domain by changing the latitude range it is executed in, using the same TOPMODEL parameters for the entire global domain. Next, in the updated model version, peatland extent was limited to areas with sufficient warm season precipitation, which has been shown to play an important role in the timing of peatland formation in the West Siberian Lowlands (13). This was done by adding a condition that peatlands can only exist in areas where $P > 0.7$ PET, with P precipitation and PET potential evapotranspiration, both as annual totals summed over all days with temperatures > 0°C. Then, we reformulated the C flux from acrotelm to catotelm, F_{AC} (16), making it a function of acrotelm decomposition, as the old formulation was inconsistent. The reformulated acrotelm to catotelm flux is $F_{AC} = \gamma(R_{A,o}+R_{A,a})$, with γ =0.23 the fraction of actrotelm respiration that enters the catotelm. Finally, peat decomposition rates were slightly re-calibrated, as the original parameters yielded unrealistically high C stocks in tropical peatlands. This was done by scanning the parameter space to determine values for γ and k_C that simultaneously fulfill constraints on tropical and northern peat accumulation rates (26), slightly increasing the catotelm decomposition rate constant from $k_C = 3.35x10^{-5}yr^{-1}$ to $k_C = 1.0x10^{-4}yr^{-1}$ (see (16) for equations), while the new formulation for F_{AC} has a similar effect to an increase in the catotelm formation rate constant k_P of the old model formulation. This modification leads to small changes in the pre-industrial peat accumulation rates in northern peatland areas in comparison to the parameter values used in (16), while tropical peat accumulation rates decrease to values generally in line with (2).

The peatland model was driven with orbital changes, CO_2 concentrations derived from ice-core data, and ice sheet extent determined using an ice-sheet enabled version of the CLIMBER2 model (11). The model was initialized with a 5000 year spinup period under early Eemian boundary conditions at 126 ka BP and subsequently run transiently from 126 ka BP until 0 BP. The spinup period has little effect on the simulation of peatland areas during early MIS 5e (Fig. S5, discussion below); however, MIS 5e peatland C stocks must be cautiously interpreted before 120 ka as they still contain a drift from the model spinup.

Model evaluation and error.

We evaluate the model performance for representation of peatlands in the past by comparing the timing of peat initiation in the model and dataset (Fig. S6), peat presence in the observation, model results, and both (Fig. S5), and the magnitude of present-day peatland extent and C stocks between the model and previous estimates (Table S3). However, the estimates of present day peatland area distributions differ widely among

studies, resulting in considerable uncertainty in peatland extent (14). Similar caveats apply to existing estimates of peatland carbon stocks (Table S3).

The global peatland model reproduces the location of present-day peatlands reasonably well. The model underestimates large peatland areas and overestimates small areas. It does, however, capture the most important peatland regions, including the West Siberian Lowlands, Hudson Bay Lowlands, and Finland (7). Similar caveats apply to tropical peatland areas, where the model captures the major peat forming regions of Southeast Asia, the Congo, and the Amazon (Fig. S6). Modeled northern peatland areas are likely underestimated by the global peatland model compared with previous estimates (Table S3). However, areas of complex terrain, especially mountainous areas, are excluded from the model domain for technical reasons, despite known occurrences of peat in these areas, which may account for some of the underestimation. This particularly affects peatland distribution in Alaska, Western Canada, and Scandinavia, where peatlands largely exist in mountainous areas. Modeled tropical areas are well within the range of existing estimates, which vary by more than a factor of four (Table S3). Comparing the timing of peatland development between the model and the observations showed that the model initiates peat too early in much of the West Siberian Lowlands (Fig. S6). The bias towards older peatlands in this larger region may explain why total northern C stocks are similar to previous estimates while there is some discrepancy between modeled peatland areas and independent estimates (Table S3), as well as between C accumulation rates and observations as discussed below. A comparison between the modeled peatland extent and observations for both warmer and cooler periods shows reasonably good agreement in terms of capturing the major peatland areas (Fig. S5). During warm periods such as MIS 5e and MIS 3, model results showed extensive peatland area across much of Eastern North America, West Siberian Lowlands, Indonesia, and the upper Amazon River Basin, where observations of buried peats also occur (Fig. S5). During the LGM, both model and observations show a decrease in peatland extent (Table 1, Fig. S5), with major peatland areas occurring in Indonesia, the upper Amazon River Basin, and the Southeastern U.S., where observations of buried and present-day peatlands also occur (Fig. S5). Interestingly, peat formation during warm periods in the past occurred in many of the same regions as today (Figs. S1, S5, Table S2).

In addition to uncertainties in peatland area, uncertainties in peat C accumulation rates can also result in uncertainties in peat C stocks (e.g. 2). Carbon accumulation is the net result of ecosystem productivity minus the decomposition of peat. In this study, peat decomposition rates were calibrated to achieve a best match to observed peat accumulation rates (2, 7), but since very few observations are available for tropical peatlands, the uncertainty in model calibration remains large. To evaluate uncertainties in C stocks resulting from C accumulation rates, we compare the median modeled C accumulation rates for northern latitudes against observations (17) for the corresponding time periods. The modeled C accumulation rates (mean = 15.6 g C m^{-2} y^{-1}) were significantly lower than the observations (22.5 g C m^{-2} y^{-1}) from 12.5 ka to 1 ka (Welch Two sample t-test, t=7.8, d.f.=44.0, $P<0.0001$). Differences are likely due, in part, to the spatial differences in sampling (global in the model, points in the dataset), and relatively stable climatic conditions during the Holocene in the climate forcing that doesn't reflect regional variations in temperature such as the Holocene Thermal Maximum (Fig. 2b,c).

An underestimation of apparent C accumulation rates by as much as 45% could result in an underestimation of C stocks of a similar magnitude.

Error due to uncertainties in peatland areas and peat C accumulation rates are the two major contributors to the uncertainty in our modeled present-day peatland C stocks. By propagating this error for our model results, we estimate the range of uncertainty in our present-day modeled peatland C stock (Table S3). With the uncertainty, our estimated C stocks fall within the uncertainty range of other previous studies. Given the lack of information about the areal extent of wetlands during the past, we assume that the errors in present-day peatland area scale with peatland area (7) and that our modeled northern peatland area in the past is overestimated by ~25% or underestimated by as much as 40%, while uncertainty in our tropical peatland area is 50% to 70% (Table S3).

Accounting for error in both area and C accumulation rates results in present-day C stocks of 452 to 822 Pg C in northern peatlands and 113 to 296 Pg C in tropical peatlands. These upper estimates are significantly larger than any other estimates of either northern or tropical peatland C stocks using either the mean C density approach or the time-varying rate of C accumulation (Table S3). Therefore, using the other, independent estimates of present-day peatland C stocks, we reject the maximum uncertainty in peat C stocks resulting from underestimating both the maximum value of peatland areas and peatland C accumulation rates as exceeding the constraints imposed by the data. These independent constraints imply that the combined uncertainty in modeled peatland C stocks due to peatland areas and C accumulation rates cannot exceed the maximum uncertainty for one factor alone. Therefore, we approximate the upper uncertainty limit with the maximum of the two uncertainties (upper limit, C accumulation rate) and use this for the upper uncertainty limit of peat C stocks throughout time (Table S3). Unfortunately, there were no measurements made during most of the Holocene with which to compare our modeled peatland C stocks, so we assume that the error scales linearly with modeled C stocks.

We compared the modeled C stocks with the observed peatland extent, as determined by the number of sites with active peat deposition (Fig. S7). The temporal variation in northern peatland extent agreed well with modeled C stocks using a visual comparison (Fig. S7a, b), although the magnitude differed between the two quantities due to biases in observations including sample preservation, sample discovery, and difficulties dating sediments with ages beyond the limits of radiocarbon dating (~45 ka). In the tropics, there was substantial temporal variability in peatland extent, but no trends in peatland extent during the past 50 ka (Fig. S7c), in good agreement with modeled tropical peat C stocks (Fig. S7d).

We used Spearman's rank correlation to compare between observations of northern peatland extent and modeling results of northern C stocks for the pre-LGM periods. Spearman's rank correlation is non-parametric test that shows a correlation between the ranked values of each quantity (here the modeled C stocks and number of active sites), indicating whether these variables vary monotonically. While we expect a correlations between these quantities, we do not expect the correlations to be linear because of the constraints of the observational dataset including factors such as bias in the preservation of buried peat deposits, bias in the discovery of buried peat deposits, and the difficulties of assigning ages to deposits older than ~45 ka due to the limits of radiocarbon dating. We implemented the rank correlation in R Statistical Software (18) between modeled C

stocks and the number of active sites for each of the periods in Table 1 using the command "cor.test" with the method set to Spearman. We repeated this analysis for the post-LGM periods using the modeled C stocks and the sum of present-day and buried peats for the periods in Table 2 but instead used the Pearson's method for linear correlation because of the more robust dataset during these periods.

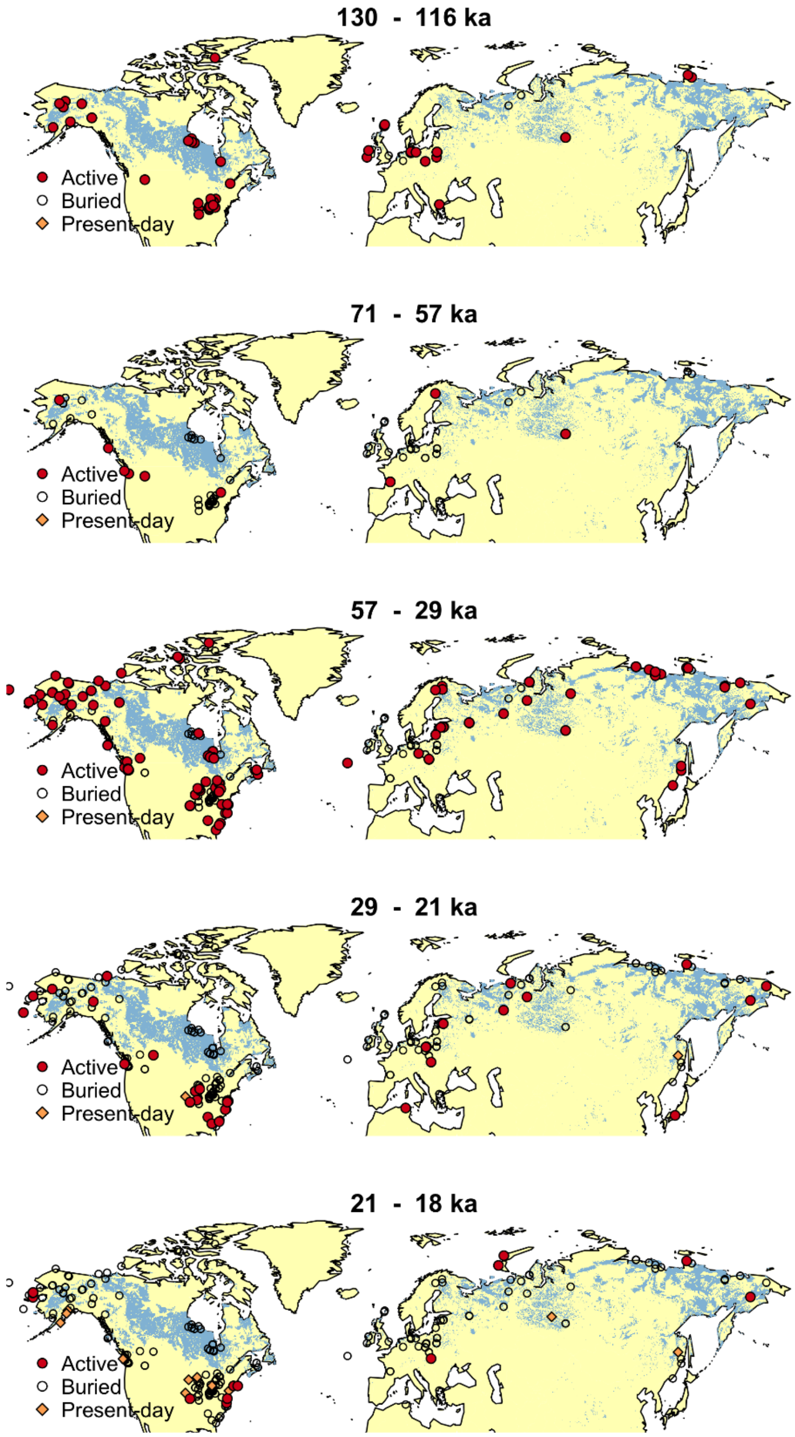

Fig. S1. Location and timing of peatland occurrence in northern latitudes (> 40° N) from the last interglacial (MIS 5e, 130 – 116 ka) to the LGM (21 – 18 ka). Red circles represent active peat deposition during the period shown, while open circles indicate the existence of a buried peat deposit. Present-day peatlands are shown by orange diamonds; present-day peatland extent is shown in blue (2).

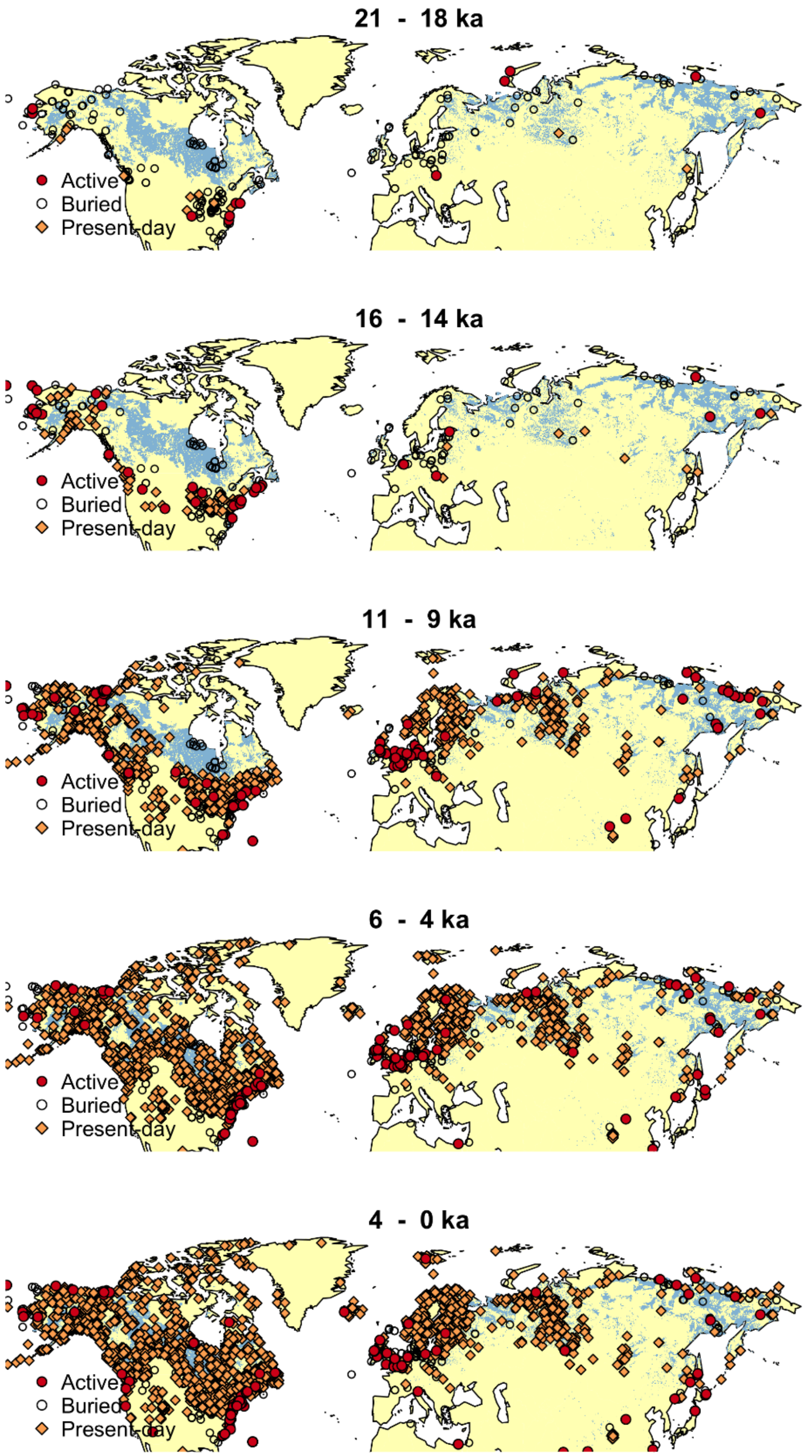

Fig. S2. Location and timing of peatland occurrence in northern latitudes (> 40° N) from the LGM (21 – 18 ka) to the pre-industrial period. Red circles represent active peat deposition during the period shown, while open circles indicate the existence of a buried peat deposit. Present-day peatlands are shown by orange diamonds; present-day peatland extent is shown in blue (2).

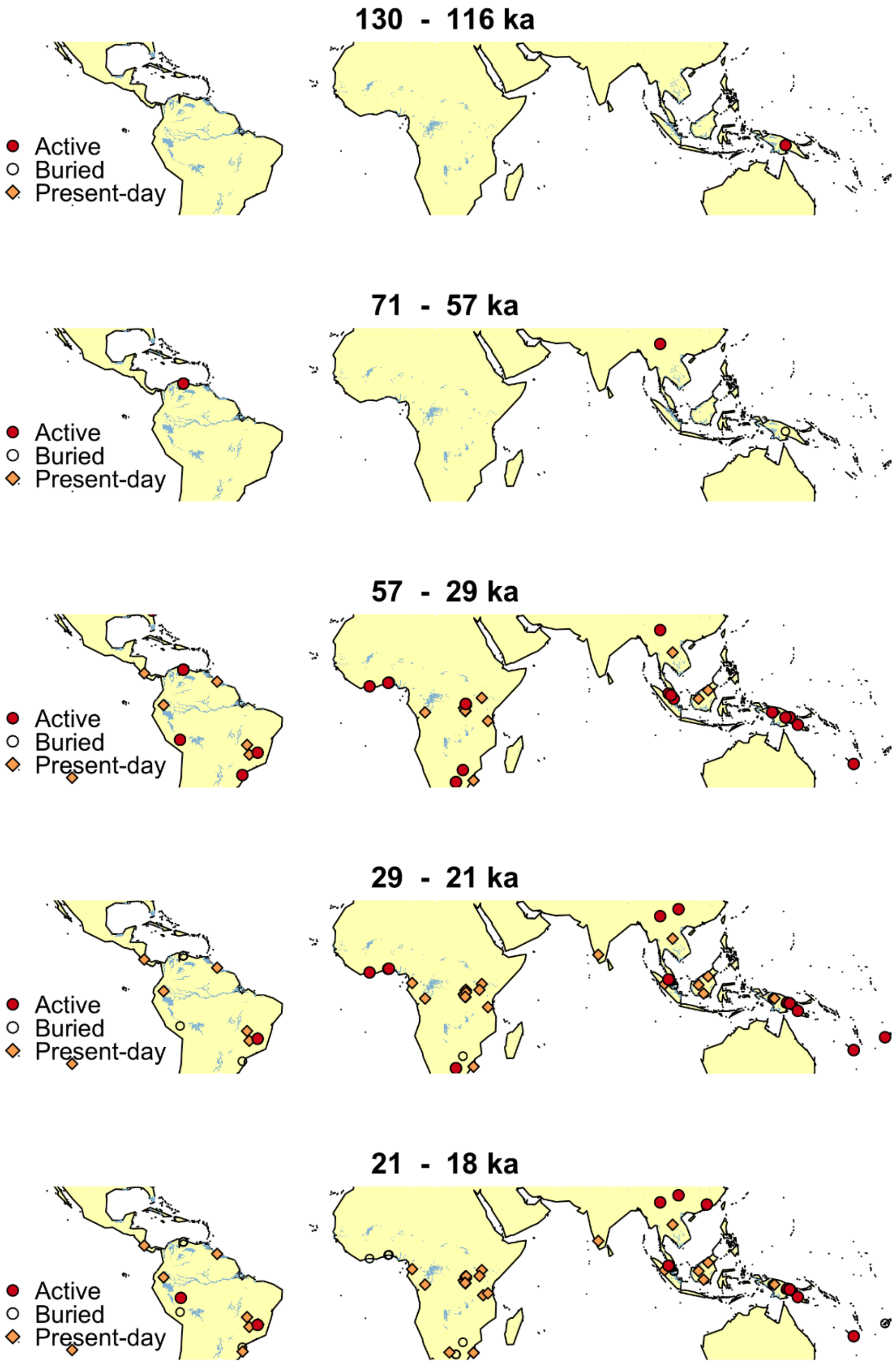

Figure S3. Location and timing of peatland occurrence in tropical latitudes (30° N – 30° S) from the last interglacial (130 – 71 ka) to the LGM (21 – 18 ka). Red circles represent active peat deposition during the period shown, while open circles indicate the existence of a buried peat deposit. Present-day peatlands are shown by orange diamonds; present-day peatland extent is shown in blue (2).

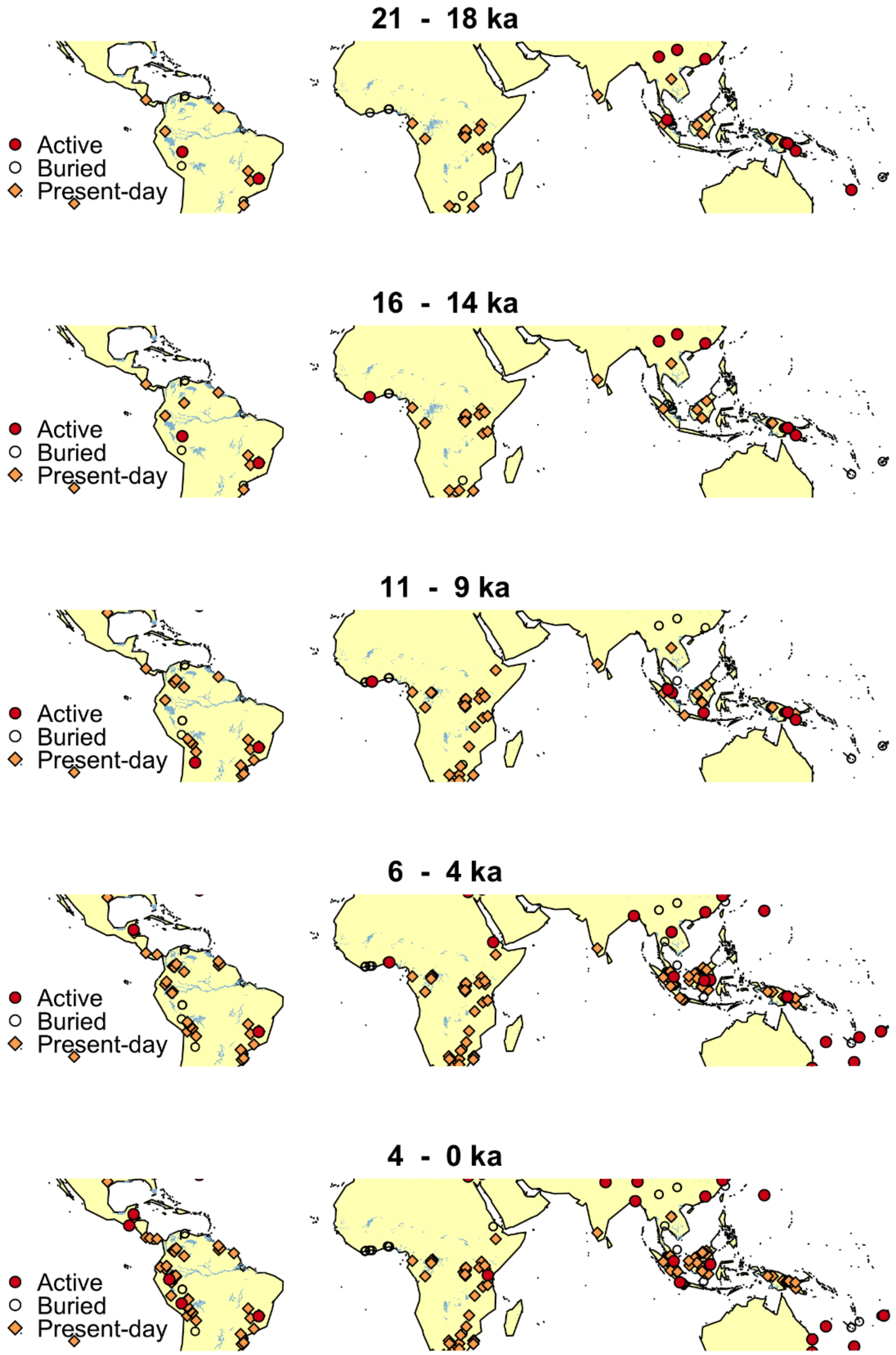

Figure S4. Location and timing of peatland occurrence in in tropical latitudes (30° N – 30° S) from the LGM (21 – 18 ka) to the pre-industrial period. Red circles represent active peat deposition during the period shown, while open circles indicate the existence of a buried peat deposit. Present-day peatlands are shown by orange diamonds; present-day peatland extent is shown in blue (2).

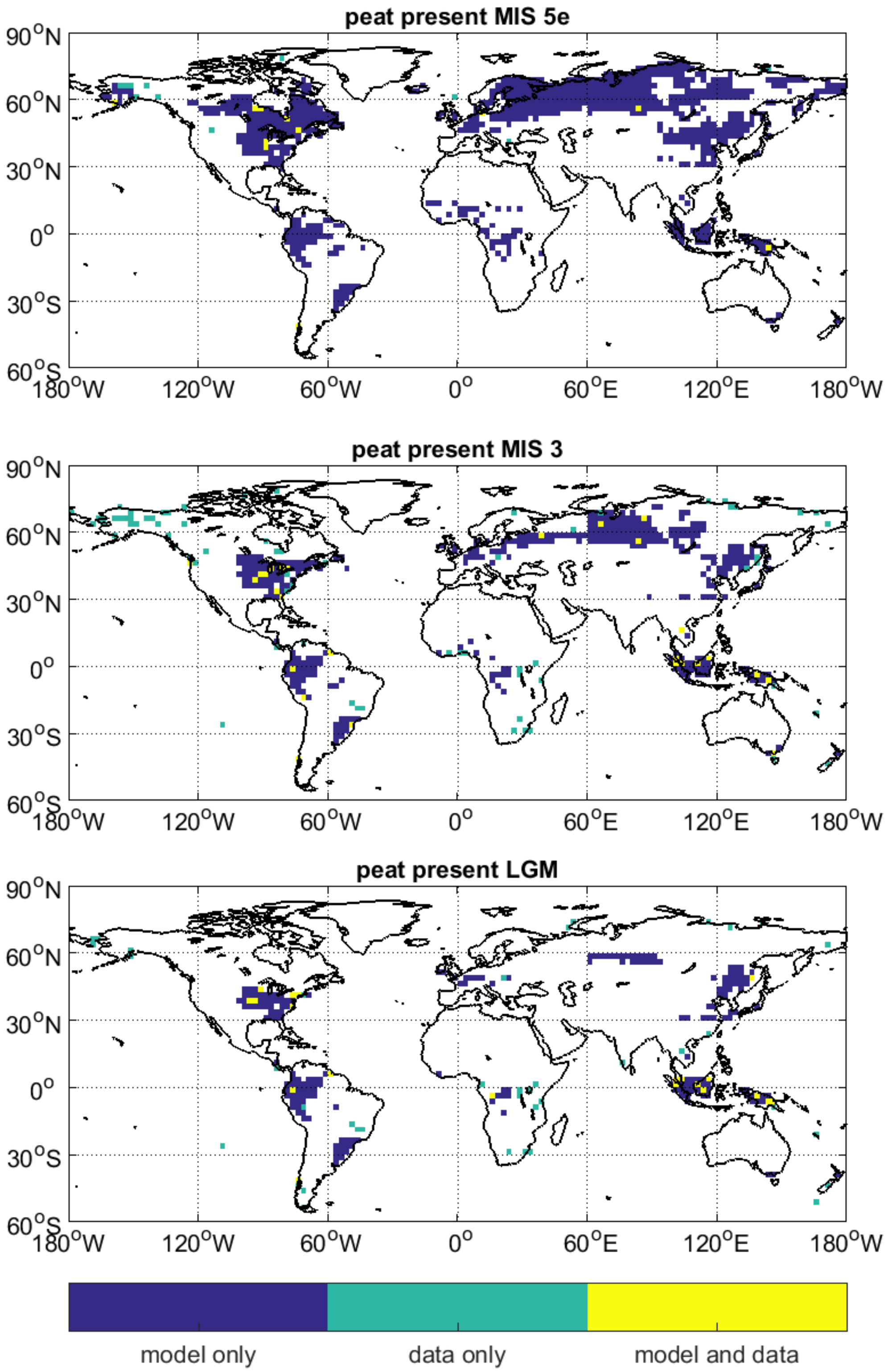

Figure S5. Presence of peat in model (blue), observations (green), and where model and data both show peatland presence (yellow). Mountainous areas were masked from the model simulation, including parts of Alaska, Western Canada, and Scandinavia.

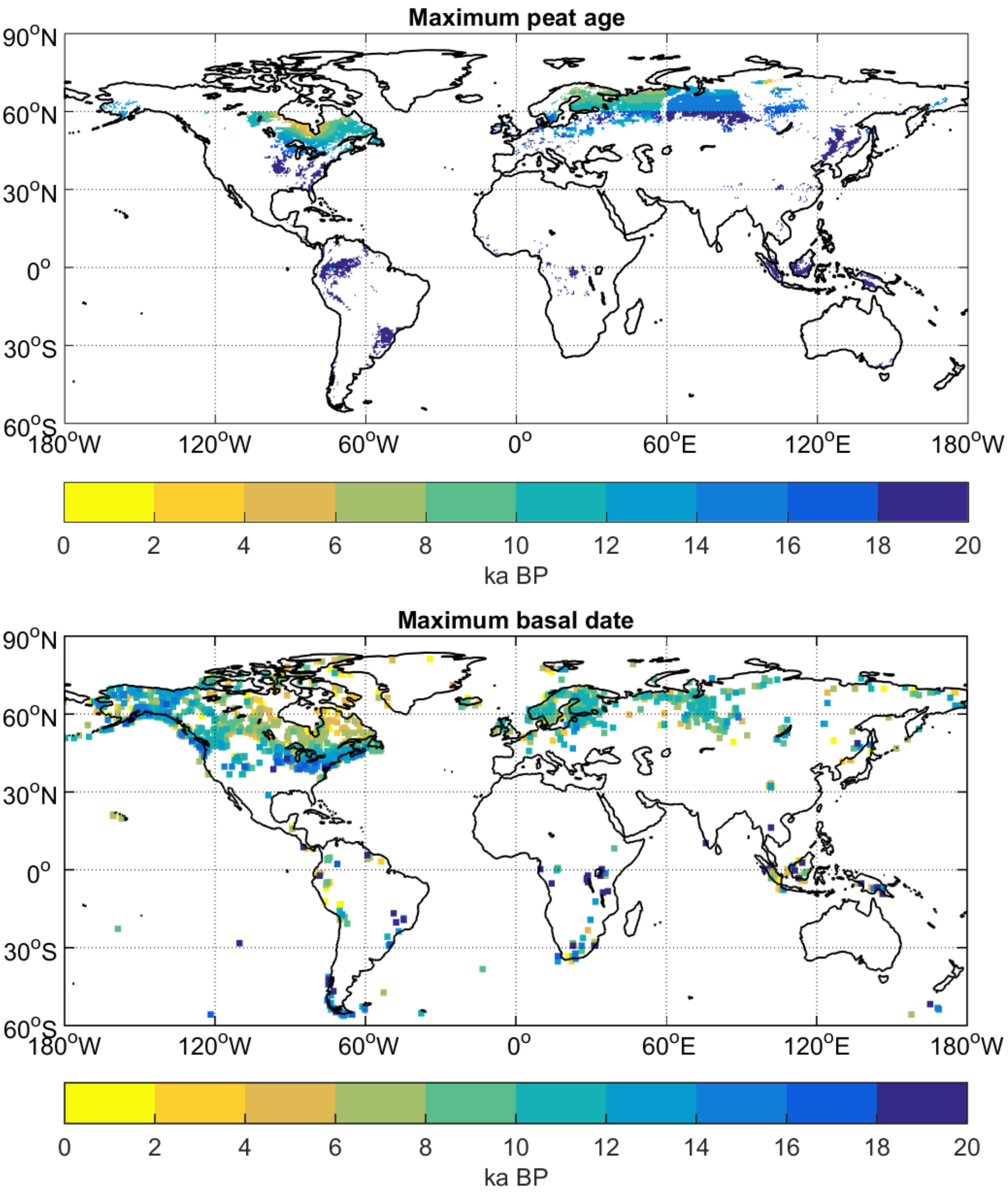

Figure S6. Timing of modeled (top) and measured (bottom) peatland initiation for global peatlands. Mountainous areas were masked from the model simulation, including parts of Alaska, Western Canada, and Scandinavia.

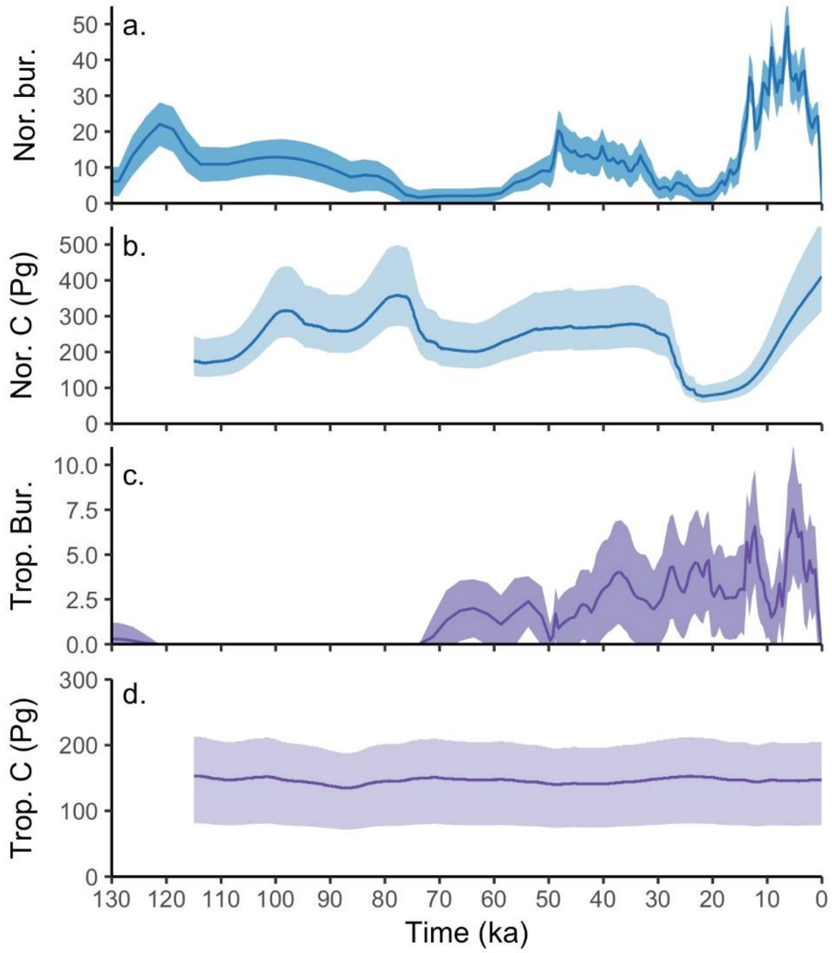

Figure S7. Peat deposition records and modeled peatland C stocks for northern (>40°N) and tropical (30°N – 30°S) peatlands for the last 130 ka. a) number of active northern peat deposits now buried (count); b) modeled northern peatland C stocks (Pg C); c) number of active peat deposits (now buried) in tropical regions (count); d) modeled tropical peatland C stocks (Pg C).

Table S1. Summary of tropical (30°S to 30° N) peatland sites and C stocks between the last interglacial (130 ka) and the pre-industrial (0.1 ka). Active sites indicates the total number of sites with active peat deposition that are now buried. Present-day indicates the number and percentage of present-day peatland sites that were established by the period of interest.

**Period**	**Age**	**Active sites**	**Present-day**		**Modeled C stock**	
	(ka)	(count)	(count)	(%)	(Pg)	
MIS 5e	130-116	1	0	0	140	(75-215)
MIS 5a-d	116-71	3	0	0	145	(75-215)
MIS 4	71-57	6	0	0	150	(80-220)
MIS 3	57 -29	26	24	12	140	(75-215)
MIS 2	29-21	16	34	17	150	(80-225)
LGM	21-18	11	37	20	150	(80-225)
Bølling-Allerød	14.7 – 12.7	17	57	30	145	(80-215)
Holocene	11.7	10	65	33	145	(75-215)
	8.2	5	96	50	145	(80-215)
Mid-Holocene	5	13	146	75	145	(80-215)
Pre-Industrial	0.1	0	197	100	145	(80-220)

Table S2. Model-derived changes in global peatland area between MIS6e, MIS3, LGM and pre-industrial. Area losses from glaciation, continental shelf flooding and other causes are expressed as percentages of the area at the previous time. Area gains from deglaciation, shelf emergence, other causes and unchanged areas are expressed as a percentage of the area at the time.

**Period**	**Age**	**Total area**	**Loss glaciation**	**Loss flooding**	**Loss other**	**Gain deglaciation**	**Gain emergence**	**Gain other**	**Unchanged**
	(ka)	(10^6 km^2)	(%)	(%)	(%)	(%)	(%)	(%)	(%)
MIS 5e	126-121	2.5	--	--	--	--	--	--	--
MIS 3	50-40	1.3	22	--	28	--	2	--	92
LGM	21-19	0.9	4	--	24	--	2	--	91
Pre-Industrial	2-0	2.2	--	5	--	32	--	28	36

Table S3. Independent estimates of present-day peatland area and C stock for northern high latitude regions and tropical regions.

Source	Area (10^3 km^2)	C stock (Pg C)
Northern peatlands		
Gorham (1)	3460	455
Joosten and Clarke (19)		270-370
Yu, Loisel, Brosseau, Beilman and Hunt (2)	4000[a]	547 (473-621)
Xu, Morris, Liu and Holden (14)	2854	
This study	1450	410 (315-590)
Tropical peatlands		
Lehner and Döll (20)	441	
Yu, Loisel, Brosseau, Beilman and Hunt (2)	368.5	50 (44-55)
Page, Rieley and Banks (21)	368.5	89 (82-92)
Gumbricht, *et al.* (22)	1700	
Dargie, *et al.* (23)		105
Xu, Morris, Liu and Holden (14)	668	
This study	684	147 (78-218)

[a]Yu et al. (2010) cite estimated areas from 3.88 to 4.09 × 10^6 km^2 by Maltby and Immirzi [1993].

Additional data table S1 (separate file)

Synthesis dataset of buried peat profiles. File includes references, location, timing of peat deposition, lithological description, description of burial type, calibrated ages, dating methodology, and availability of additional data in the original publication.

Additional data table S2 (separate file)

Synthesis dataset of peatland initiation date. File includes references, location, basal age (calibrated and uncalibrated), original references, whether data was included in a previous synthesis (e.g. Yu, Loisel, Brosseau, Beilman and Hunt (2), MacDonald, *et al.* (24), Gorham, Lehman, Dyke, Janssens and Dyke (25), Jones and Yu (26), Korhola, *et al.* (27), Dommain, Couwenberg and Joosten (28), Packalen, Finkelstein and McLaughlin (29)).

References

1. Gorham E (1991) Northern Peatlands - Role in the Carbon-Cycle and Probable Responses to Climatic Warming. *Ecological Applications* 1(2):182-195.
2. Yu Z, Loisel J, Brosseau DP, Beilman DW, & Hunt SJ (2010) Global peatland dynamics since the Last Glacial Maximum. *Geophys. Res. Lett.* 37(13):L13402.
3. Frolking S, Talbot J, & Subin ZM (2014) Exploring the relationship between peatland net carbon balance and apparent carbon accumulation rate at century to millennial time scales. *The Holocene*.
4. Ise T, Dunn AL, Wofsy SC, & Moorcroft PR (2008) High sensitivity of peat decomposition to climate change through water-table feedback. *Nature Geosci* 1(11):763-766.
5. Staff SS (1999) Soil taxonomy: A basic system of soil classification for making and interpreting soil surveys. in *U.S. Department of Agriculture Handbook*.
6. Brigham-Grette J (1989) Geochronology. *Late Cenozoic History of the Interior Basins of Alaska and the Yukon*, eds Carter LD, Hamilton TD, & Galloway JP (U.S. Geological Survey), p 107.
7. Kleinen T, Brovkin V, & Schuldt RJ (2012) A dynamic model of wetland extent and peat accumulation: results for the Holocene. *Biogeosciences* 9(1):235-248.
8. Sitch S, *et al.* (2003) Evaluation of ecosystem dynamics, plant geography and terrestrial carbon cycling in the LPJ dynamic global vegetation model. *Global Change Biology* 9(2):161-185.
9. Petoukhov V, *et al.* (2000) CLIMBER-2: a climate system model of intermediate complexity. Part I: model description and performance for present climate. *Climate dynamics* 16(1):1-17.
10. Kleinen T, Brovkin V, von Bloh W, Archer D, & Munhoven G (2010) Holocene carbon cycle dynamics. *Geophysical Research Letters* 37(2).
11. Ganopolski A, Calov R, & Claussen M (2010) Simulation of the last glacial cycle with a coupled climate ice-sheet model of intermediate complexity. *Clim Past* 6(2):229-244.
12. Spahni R, Joos F, Stocker BD, Steinacher M, & Yu ZC (2013) Transient simulations of the carbon and nitrogen dynamics in northern peatlands: from the Last Glacial Maximum to the 21st century. *Clim. Past* 9(3):1287-1308.

13. Alexandrov GA, Brovkin VA, & Kleinen T (2016) The influence of climate on peatland extent in Western Siberia since the Last Glacial Maximum. *Scientific Reports* 6:24784.
14. Xu J, Morris PJ, Liu J, & Holden J (2018) PEATMAP: Refining estimates of global peatland distribution based on a meta-analysis. *CATENA* 160:134-140.
15. Brook EJ, Harder S, Severinghaus J, Steig EJ, & Sucher CM (2000) On the origin and timing of rapid changes in atmospheric methane during the Last Glacial Period. *Global Biogeochemical Cycles* 14(2):559-572.
16. Fairbanks RG (1989) A 17,000-year glacio-eustatic sea-level record- Influence of glacial melting rates on the Younger Dryas event and deep-ocean circulation. *Nature* 342(6250):637-642.
17. Loisel J*, et al.* (2014) A database and synthesis of northern peatland soil properties and Holocene carbon and nitrogen accumulation. *The Holocene* 24(9):1028-1042.
18. Team RDC (2008) R: A language and environment for statistical computing (R Foundation for Statistical Computing, Vienna, Austria).
19. Joosten H & Clarke D (2002) *Wise use of mires and peatlands* (International mire conservation group).
20. Lehner B & Döll P (2004) Development and validation of a global database of lakes, reservoirs and wetlands. *Journal of Hydrology* 296(1-4):1-22.
21. Page SE, Rieley JO, & Banks CJ (2011) Global and regional importance of the tropical peatland carbon pool. *Global Change Biology* 17(2):798-818.
22. Gumbricht T*, et al.* (2017) An expert system model for mapping tropical wetlands and peatlands reveals South America as the largest contributor. *Global Change Biology* 23(9):3581-3599.
23. Dargie GC*, et al.* (2017) Age, extent and carbon storage of the central Congo Basin peatland complex. *Nature* 542(542):96-90.
24. MacDonald GM*, et al.* (2006) Rapid early development of circumarctic peatlands and atmospheric CH4 and CO2 variations. *Science* 314(5797):285-288.
25. Gorham E, Lehman C, Dyke A, Janssens J, & Dyke L (2007) Temporal and spatial aspects of peatland initiation following deglaciation in North America. *Quaternary Science Reviews* 26(3-4):300-311.
26. Jones MC & Yu Z (2010) Rapid deglacial and early Holocene expansion of peatlands in Alaska. *Proceedings of the National Academy of Sciences* 107(16):7347-7352.
27. Korhola A*, et al.* (2010) The importance of northern peatland expansion to the late-Holocene rise of atmospheric methane. *Quaternary Science Reviews* 29(5–6):611-617.
28. Dommain R, Couwenberg J, & Joosten H (2011) Development and carbon sequestration of tropical peat domes in south-east Asia: links to post-glacial sea-level changes and Holocene climate variability. *Quaternary Science Reviews* 30(7–8):999-1010.
29. Packalen MS, Finkelstein SA, & McLaughlin JW (2014) Carbon storage and potential methane production in the Hudson Bay Lowlands since mid-Holocene peat initiation. *Nat Commun* 5.
